# Elucidation of Secondary Structure and Toxicity of α-Synuclein Oligomers and Fibrils Grown in the Presence of Phosphatidylcholine and Phosphatidylserine

**DOI:** 10.1021/acschemneuro.3c00314

**Published:** 2023-08-21

**Authors:** Tianyi Dou, Mikhail Matveyenka, Dmitry Kurouski

**Affiliations:** † Department of Biochemistry and Biophysics, Texas A&M University, College Station, Texas 77843, United States; ‡ Department of Biomedical Engineering, Texas A&M University, College Station, Texas 77843, United States

**Keywords:** α-synuclein aggregation, AFM-IR, protein−lipid interactions, cytotoxicity, protein secondary structure, phosphatidylcholine, and phosphatidylserine

## Abstract

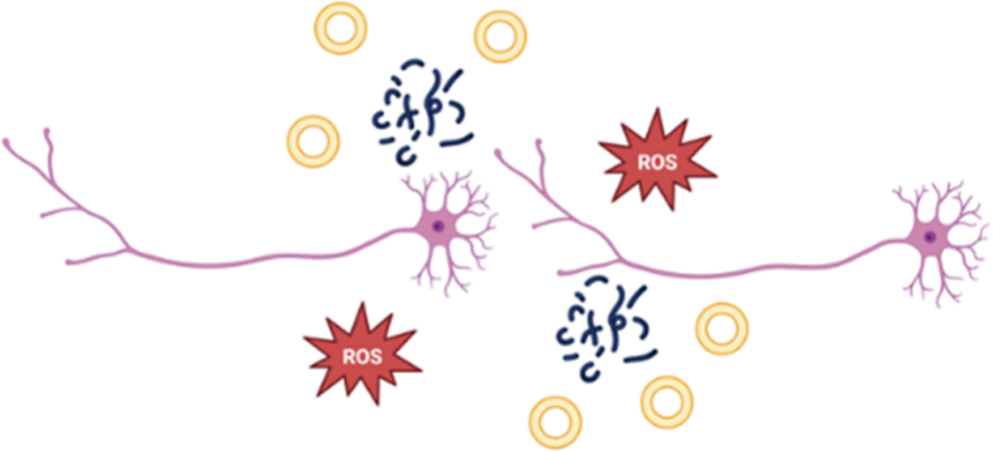

Abrupt aggregation of α-synuclein (α-Syn) in the midbrain hypothalamus and thalamus is a hallmark of Parkinson’s disease (PD), the fastest growing neurodegenerative pathology, projected to strike 12 million people by 2040 worldwide. In this study, we examine the effect of two phospholipids that are present in neuronal membranes, phosphatidylcholine (PC) and phosphatidylserine (PS), on the rate of α-Syn aggregation. We found that PS accelerated α-Syn aggregation, whereas PC strongly inhibited α-Syn aggregation. We also utilized the nano-infrared imaging technique, also known as atomic force microscopy infrared (AFM-IR) spectroscopy, to investigate whether PC and PS only change the rates or also modify the secondary structure of α-Syn aggregates. We found that both phospholipids uniquely altered the secondary structure of α-Syn aggregates present at the lag and growth phase, as well as the late stage of protein aggregation. In addition, compared to the α-Syn aggregates formed in the lipid-free environment, α-Syn:PC and α-Syn:PS aggregates demonstrated higher cellular toxicity to N27 rat neurons. Interestingly, both α-Syn:PC and α-Syn:PS aggregates showed similar levels of oxidative stress, but α-Syn:PC aggregates exhibited a greater degree of mitochondrial dysfunction compared to α-Syn:PS aggregates.

## Introduction

Parkinson’s disease (PD) is the second-most common neurodegenerative disease among senior people over 65 years old.^1^ Clinical studies of PD reveal the presence of Lewy bodies (LBs), extracellular formations that appear in the midbrain, hypothalamus, and thalamus.^2−4^ LBs are composed of α-synuclein (α-Syn) aggregates and fragments of cell membranes.^5,6^ α-Syn is a 14 kDa protein that regulates neurotransmitter release by synaptic vesicles.^7−10^ Although the exact cause of abrupt aggregation of α-Syn is unclear,^11,12^ the onset and spread of PD is linked to α-Syn oligomers and fibrils, protein species that express high levels of cytotoxicity.^13−16^

The utilization of cryo-electron microscopy helped to determine the secondary structure of α-Syn fibrils.^17−21^ It was found that these fibrils have the cross-β-sheet structure in which two β-sheets that are held together by hydrogen bonding stretch microns in length.^17,22−24^ However, due to the inherent heterogeneity in α-Syn aggregation, cryo-EM is currently limited in its ability to study the detailed structures of α-Syn oligomers.^25−27^ Using high-resolution AFM, Ruggeri et al. explained the mechanism of early assembly of monomeric α-Syn into elongated aggregates.^28^ Zhou and Kurouski demonstrated that aggregation of α-Syn results in at least three different classes of protein oligomers. The first class is dominated by α-helix and unordered protein, whereas the second and third classes have predominantly parallel and anti-parallel β-sheet secondary structures.^25^ Using protein lyophilization, Chen and co-workers were able to isolate stable α-Syn oligomers that had cylindric shape.^29^ The researchers found that these cylindrical oligomers possessed significantly higher cell toxicity compared to mature α-Syn fibrils.

The presence of lipid membranes in LBs suggested that lipids can be involved in α-Syn aggregation. Furthermore, it was found that α-Syn interacts with lipids forming a protein–lipid complex.^30−33^ Such complexes are held by electrostatic interactions that are taken place between charged polar heads of phospholipids and the positively charged N-terminus of the protein. Furthermore, hydrophobic interactions between the aliphatic tails of fatty acids of lipids and the hydrophobic NAC region of α-Syn are also developed in α-Syn:lipid complexes.^30−33^ Galvagnion et al. discovered that phospholipids, the major constituents of neurons’ plasma membrane, could alter protein aggregation rates.^34−39^ Dou et al. demonstrated that phosphatidylserine (PS) and phosphatidylcholine (PC) not only altered the rates of α-Syn aggregation but also uniquely modified the secondary structure of the oligomers.^40,41^ Furthermore, both lipids were found to be included in the α-Syn oligomers formed at the early stages of protein aggregation. This conclusion was made by the structural analysis of individual α-Syn oligomers grown in the presence of PC and PS using atomic force microscopy infrared (AFM-IR) spectroscopy.^40^ AFM-IR is a modern analytical technique that is capable of probing thermal expansions in individual oligomers and fibrils.^42−44^ For this, a metalized scanning probe is positioned above the sample of interest that is illuminated by pulsed tunable IR light.^44−46^ IR-induced thermal expansions in the sample are recorded by the scanning probe and converted into IR spectra, which, in turn, can be used to determine the secondary structure of the analyzed protein specimen.^28,47−52^ Using AFM-IR, Rizevsky et al. found that PC, PS, and cardiolipin uniquely altered the secondary structure of insulin oligomers and fibrils.^53^ Furthermore, these lipids were found in the structure of insulin oligomers that were formed in their presence. Matveyenka et al. also found that such oligomers exerted significantly lower cell toxicity than insulin aggregates grown in the lipid-free environment.^54,55^

Expanding upon this, we investigate the extent to which PC and PS could alter the secondary structure of α-Syn aggregates formed at the lag and growth phases and late stages of protein aggregation. For this, we utilized the thioflavin T approach to determine the extent to which lipids altered the rates of α-Syn aggregation, as well as AFM-IR to examine the secondary structure of α-Syn aggregates present at the lag and growth phases of protein aggregation, as well as mature α-Syn fibrils. Finally, we employed a set of biochemical methods to establish the connection between the structure and toxicity of α-Syn grown in the presence of lipids and in a lipid-free environment.

## Results and Discussion

In the lipid-free environment, α-Syn aggregates exhibit a well-defined lag-phase (*t*_lag_ = 12.9 ± 0.52 h) that is followed by a rapid increase in the ThT intensity, which indicates the formation of protein aggregates, Figure S1. We found that PS shortened the *t*_lag_ of α-Syn aggregation when present in equimolar concentrations with the protein. Specifically, α-Syn:PS *t*_lag_ was found to be 10.0 ± 0.76 h. At the same time, we found that PC strongly inhibited protein aggregation, Figure S1.

**Figure 1:**
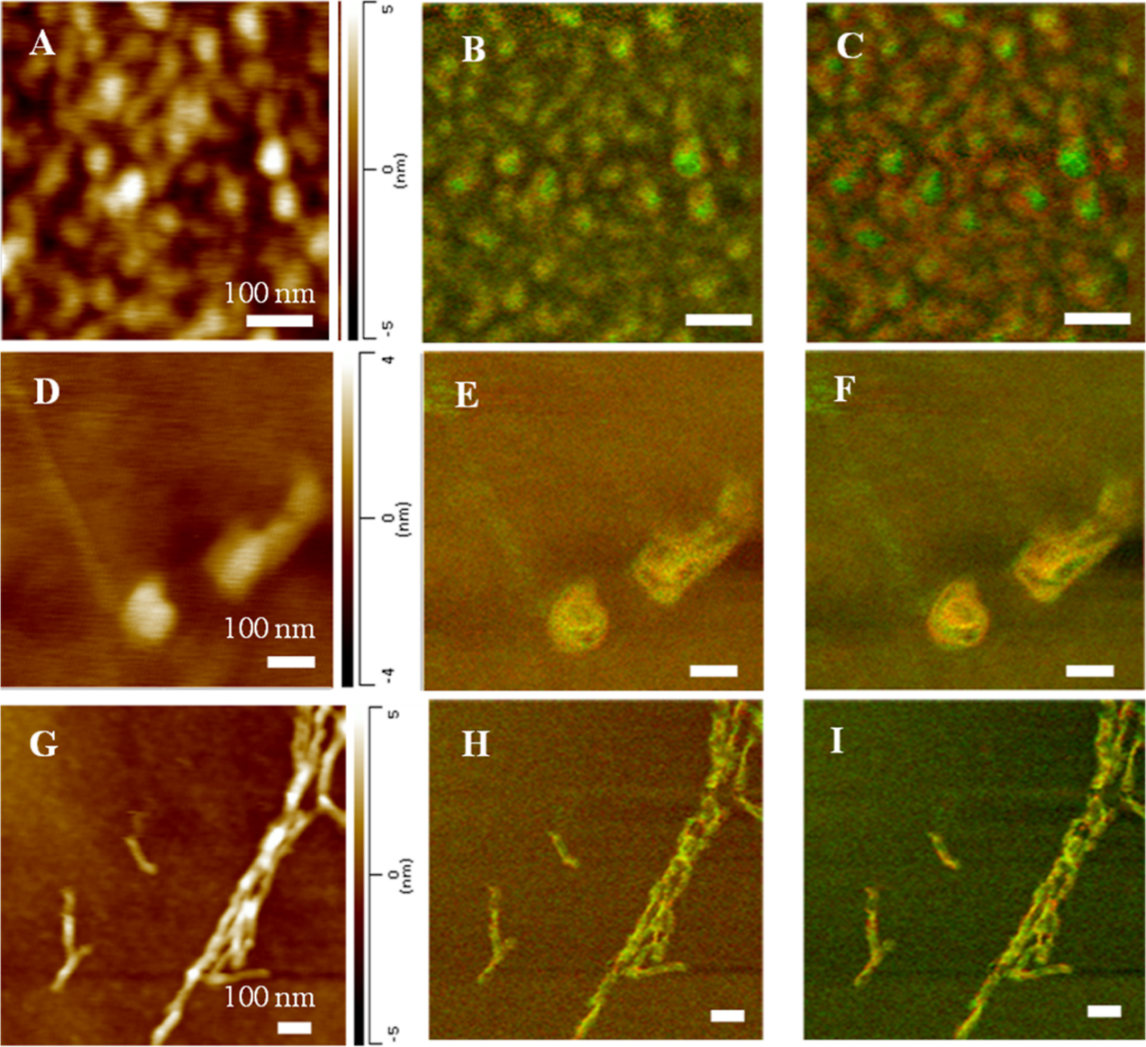
AFM-IR images of α-Syn aggregates were observed at the lag phase (A–C), growth phase (D–F), and late stage (G–I) of protein aggregation. Overlaid IR maps at 1624 cm^–1^ (green) and 1655 (red) cm^–1^ (B,E,H), as well as 1624 cm^–1^ (green) and 1694 (red) cm^–1^ (C,F,I). Scale bars are 100 nm.

Using AFM, we found that α-Syn oligomers present at the lag phase of protein aggregation in the lipid-free environment had a spherical appearance. These aggregates were ∼20 nm in diameter and had a height of around 5 nm (Figures 1A and S2). Oligomers present at the growth phase had similar spherical shapes but were larger in size compared to the oligomers observed at the lag stage. At the late stage of α-Syn aggregation, we observed fibrils that were 7–8 nm in height and ∼20 nm in width, Figure S2.

Chemical composition mapping using AFM-IR on α-Syn aggregates present at the lag phase revealed oligomers dominated by an anti-parallel β-sheet (Figure 1B,C). At the growth phase of α-Syn aggregation, we observed both oligomers and elongated protein aggregates. These species had a mixture of unordered protein secondary structures, parallel and anti-parallel β-sheets. Finally, α-Syn fibrils detected at the end of the grown phase (late stage) exhibited a parallel β-sheet secondary structure predominance.

AFM-IR imaging shows that in the presence of PC, α-Syn formed oligomers that had a height of around 3–4 nm, Figure S3. These aggregates were primarily composed of an unordered protein and anti-parallel β-sheet secondary structure with a small amount of parallel β-sheet. α-Syn:PC aggregates present at the growth stage had a very similar structure to α-Syn:PC oligomers observed at the lag phase of protein aggregation. Specifically, we observed a high amount of anti-parallel β-sheet structure with some parallel β-sheet present in these oligomers. Similar oligomers were observed at the late stage of α-Syn aggregation in the presence of PC. These results showed that PC drastically inhibits fibril formation, enabling α-Syn aggregation only into oligomeric species. This conclusion is supported by the ThT kinetics of α-Syn aggregation in the presence of PC and PS, as well as in the lipid-free environment (Figure S1).

**Figure 2:**
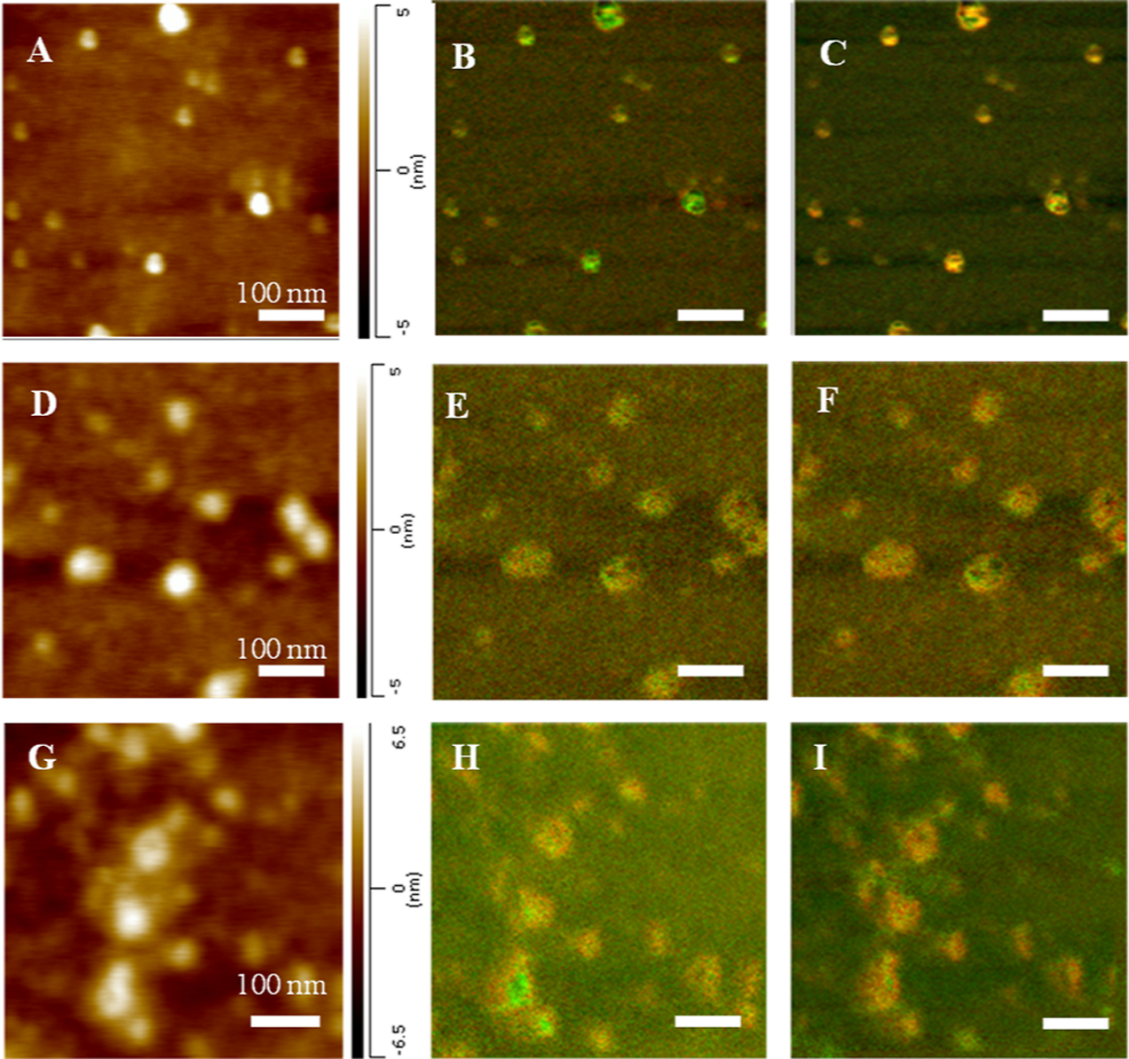
AFM-IR images of α-Syn:PC aggregates were observed at the lag phase (A–C), growth phase (D–F), and late stage (G–I) of protein aggregation. Overlaid IR maps at 1624 cm^–1^ (green) and 1655 (red) cm^–1^ (B,E,H), as well as 1624 cm^–1^ (green) and 1694 (red) cm^–1^ (C,F,I).

**Figure 3:**
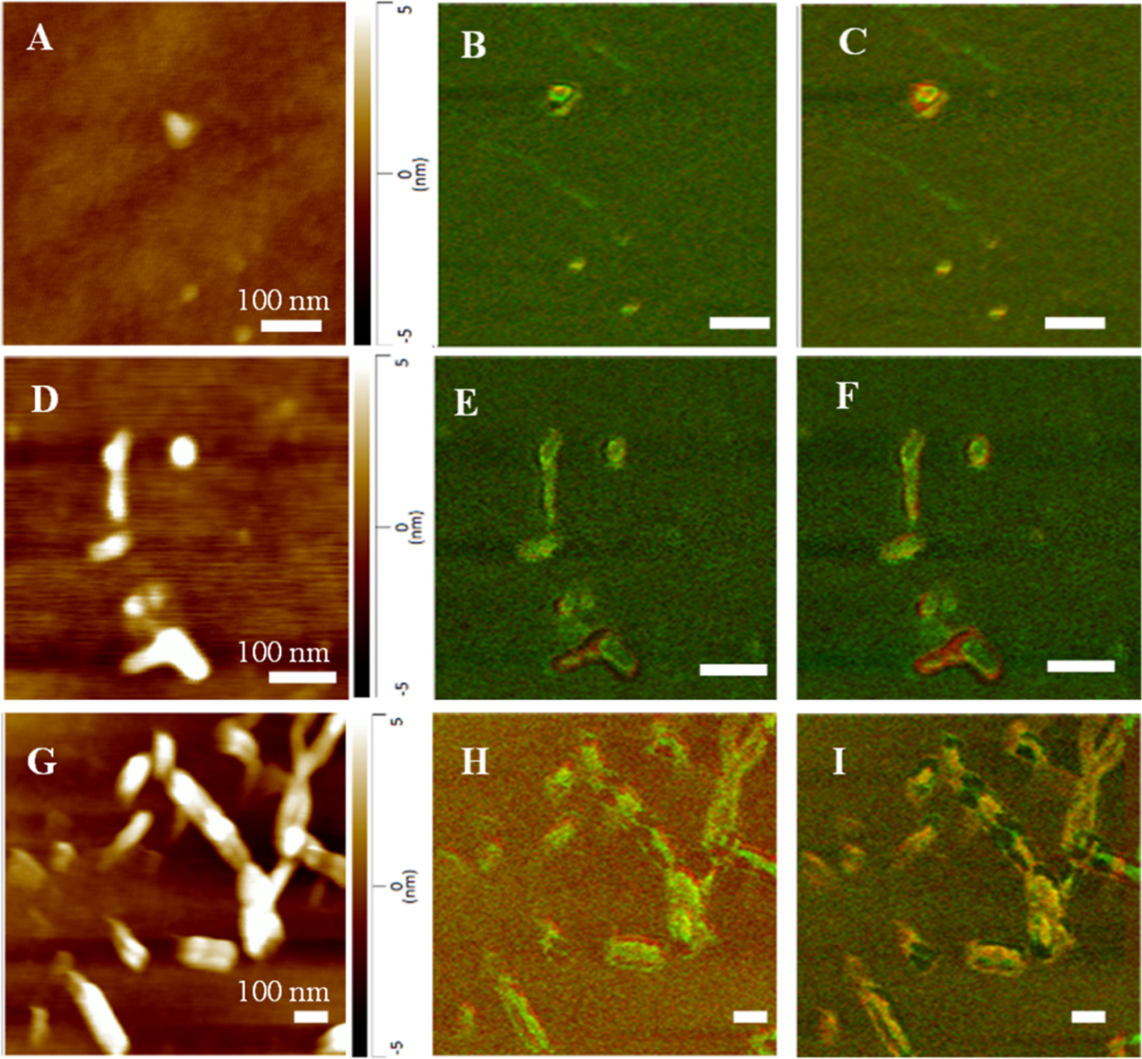
AFM-IR images of α-Syn:PS aggregates were observed at the lag phase (A–C), growth phase (D–F), and late stage (G–I) of protein aggregation. Overlaid IR maps at 1624 cm^–1^ (green) and 1655 (red) cm^–1^ (B,E,H), as well as 1624 cm^–1^ (green) and 1694 (red) cm^–1^ (C,F,I).

Oligomers formed by α-Syn at the lag phase in the presence of PS had a height of around 3–8 nm, Figure S4. These oligomers possessed a mixture of unordered protein secondary structure and parallel and anti-parallel β-sheet. We found that the size of protein aggregates observed at the growth phase of fibril formation drastically increased. Specifically, we observed 40–50 nm fibril-like structures formed by α-Syn in the presence of PS. These protein specimens had a predominantly parallel β-sheet with a small content of unordered protein secondary structure. Protein aggregates with similar dimensions and secondary structures were observed at the late stages of α-Syn aggregation in the presence of PS. These aggregates exhibited very little if any structural heterogeneity. It should be noted that fibrils grown in the presence of PS, unlike α-Syn fibrils formed in the lipid-free condition were shorter and thicker with a higher amount of anti-parallel β-sheet secondary structure (Figures 2 and 3).

**Figure 4:**
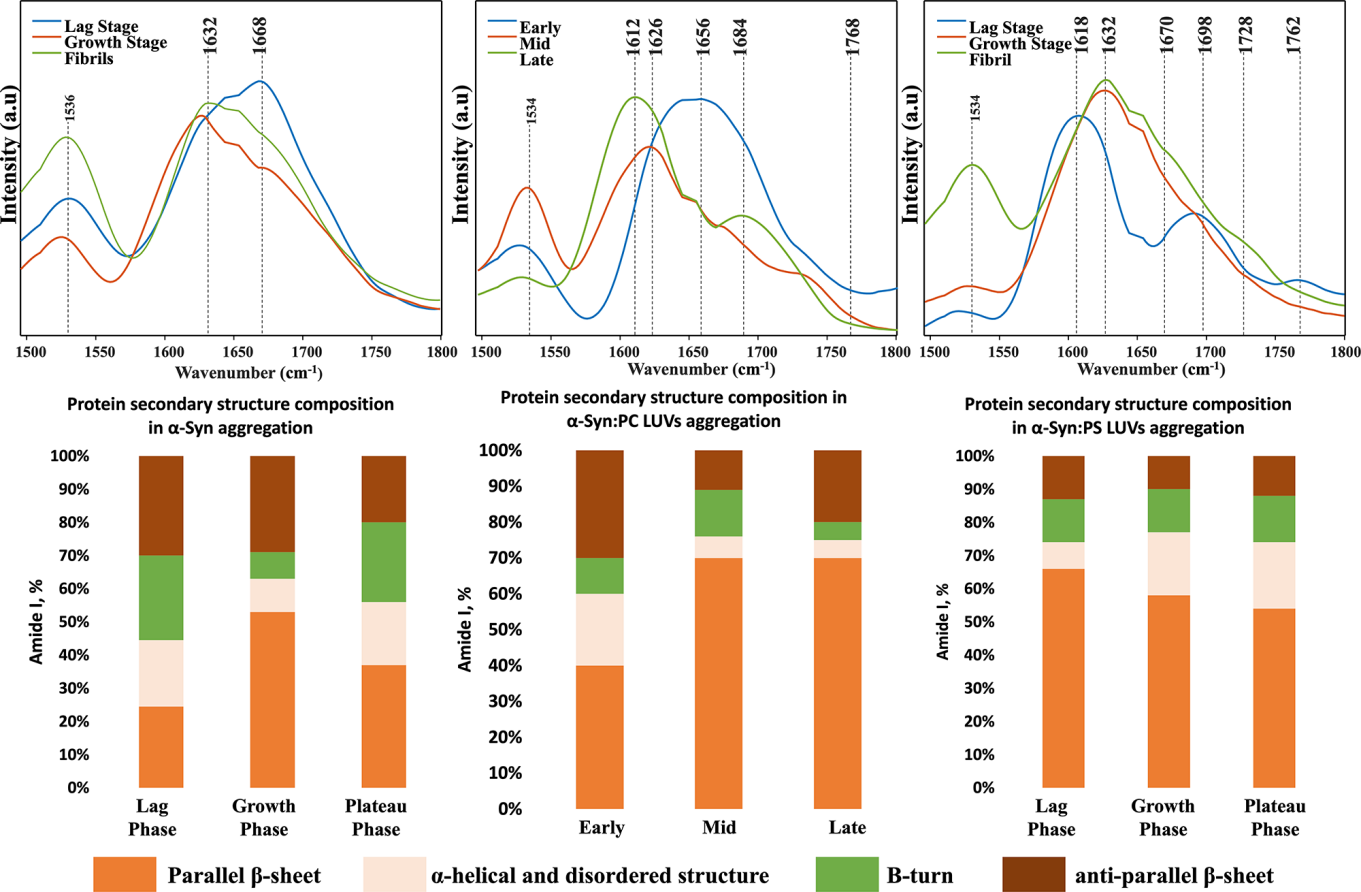
AFM-IR spectra (top) acquired from the individual aggregates grown at the lag phase (blue), growth phase (orange), and late stage (green) of protein aggregation; α-Syn (left), α-Syn:PC (center), and α-Syn:PS (right). Histograms (bottom) of the protein secondary structure composition of α-Syn (left), α-Syn:PC (center), and α-Syn:PS (right) according to the fitting of amide I region of the AFM-IR spectra. Parallel β-sheet (orange), α-helical and disordered structure (light orange), β-turn (green), and anti-parallel β-sheet (brown).

Next, we collected more than 50 spectra from individual oligomers observed at different stages of protein aggregation to provide a quantitative assessment of their secondary structure. The spectra were averaged to observe the changes in the protein secondary structure that are taken place upon α-Syn aggregation in the presence of PC and PS, as well as in the lipid-free environment, Figures 4 and S5–S7.

We found that in the lipid-free environment, aggregation of α-Syn proceeds with an increase in the amount of parallel β-sheet and a graduate decrease in the amount of anti-parallel β-sheet as oligomers formed at the lag phase propagated into fibrils. We also found a stepwise decrease in the amount of α-helical content as the oligomers observed at the lag phase propagated into higher order aggregates (growth phase), Figures S8–S11. The opposite contribution of the unordered protein secondary structure was observed. Specifically, we found that only 4% of the secondary structure of early stage oligomers was occupied with unordered protein, whereas α-Syn aggregates observed at the growth phase contained 11–13% of an unordered protein secondary structure. These conclusions are in good agreement with previously reported results by Zhou and Kurouski.^56^

In the presence of PS, drastically different transformations in the secondary structure of α-Syn aggregates were observed, as shown in Figures 4 and S5–S7. Specifically, we found a graduate decrease in the amount of parallel β-sheet content and an increase in the amount of α-helical content as oligomers propagated from the lag phase into fibrils. These findings suggest that PS interacts with the β-sheet regions of the lag-phase oligomers, limiting β-sheet-templated fibril growth. We also found that the amount of the anti-parallel β-sheet remained nearly identical in Syn:PS oligomers and fibrils present at different stages of protein aggregation, Figures S8–S11. Thus, parallel rather than anti-parallel is affected by PS. Our results showed that PS drastically altered the protein secondary structure of α-Syn oligomers compared to α-Syn oligomers grown in the lipid-free environment. Specifically, lag and middle phases α-Syn:PS aggregates possess a higher amount of parallel β-sheet and a lower amount of anti-parallel β-sheet and an unordered protein compared to α-Syn oligomers observed at the same time points of protein aggregation. At the same time, the secondary structure of α-Syn and α-Syn:PS fibrils is very similar, Figures S8–S11.

AFM-IR analysis revealed a decrease in the amount of parallel β-sheet in α-Syn:PC oligomers present at the middle stage of protein aggregation compared to α-Syn:PC oligomers observed at the lag phase, Figures S8–S11. We also observed an increase in the amount of anti-parallel β-sheet and an unordered protein secondary structure. However, α-Syn:PC oligomers observed at the late stage of protein aggregation were dominated by a parallel β-sheet with a very low amount of anti-parallel β-sheet and an unordered protein secondary structure compared to α-Syn and α-Syn:PS aggregates observed at this stage of α-Syn aggregation, Figures S8–S11. These results suggest that the structural transformation of α-Syn:PC oligomers is rather complex and highly likely to include substantial re-arrangement of protein secondary structures.

It should be noted that all the acquired AFM-IR spectra from α-Syn:PC and α-Syn:PS aggregates exhibit the vibrational band around 1720 cm^–1^, which originates from the carbonyl (C=O) vibration of lipid esters. These findings demonstrate that PC and PS are present in the α-Syn aggregates that were grown in their presence (Figures S6, S7, and S12). At the same time, this band was not visible in the AFM-IR spectra of α-Syn aggregates formed in the lipid-free environment (Figures S5 and S12). It should be noted thatα-Syn aggregates formed in the lipid-free environment possessed a substantial amount of anti-parallel β-sheet, which has a vibrational band at ∼1695 cm^–1^. This band tails down to the ∼1720 cm^–1^ spectral region, which gives a wrong impression about the presence of ∼1720 cm^–1^ in the AFM-IR spectra acquired from α-Syn aggregates formed in the lipid-free environment.

**Figure 5:**
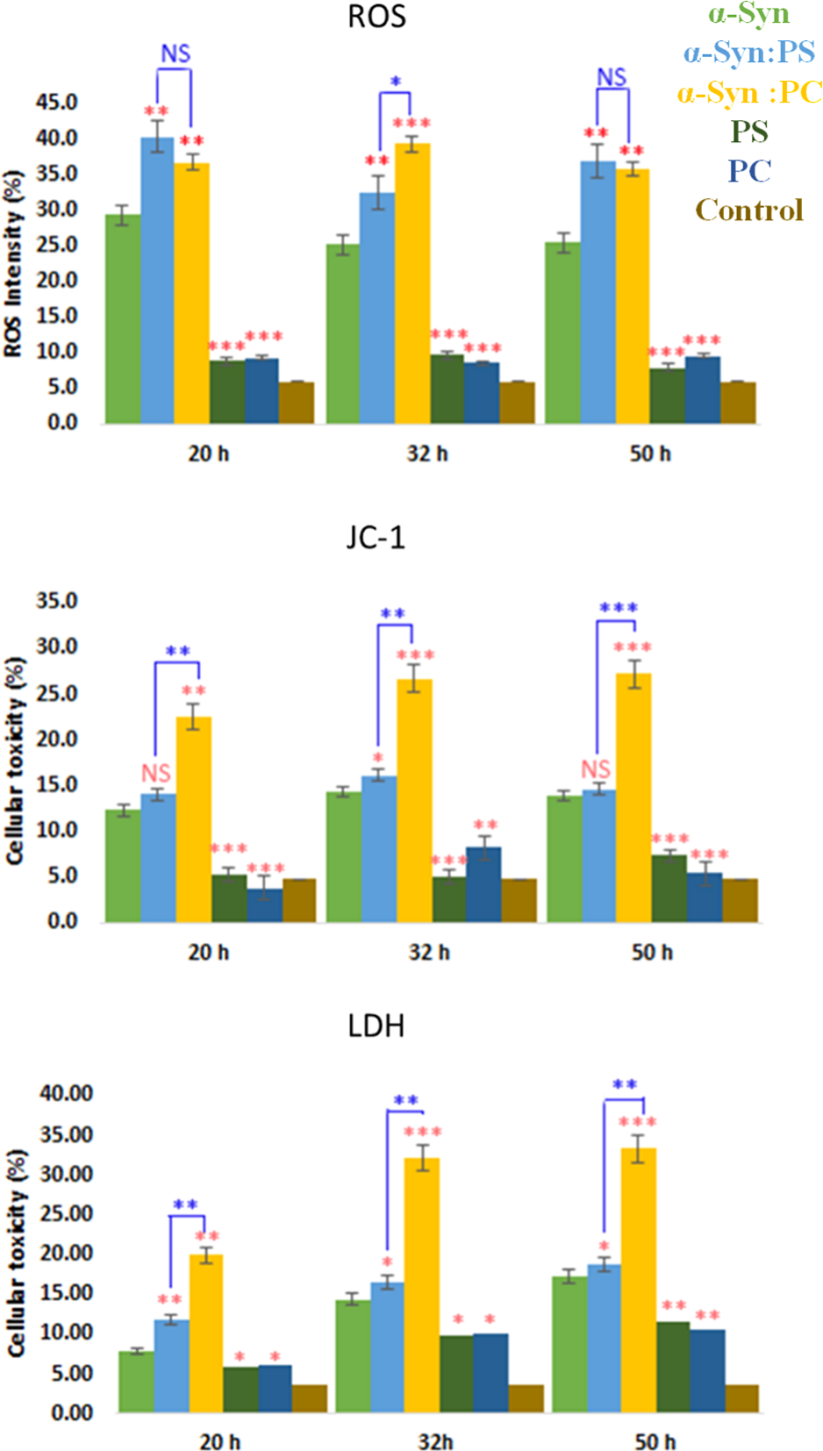
Histograms of ROS (top), JC-1(middle), and LDH (bottom) toxicity assays of α-Syn (green, 70AD47), α-Syn:PS (light blue, 5B9BD5), and α-Syn:PC (yellow, FFC000) aggregates grown at lag phase (20 h), growth phase (32 h), and late stage (50 h) of protein aggregation, as well as PC (olive, 385723) and PS (navy, 2F5597) lipids themselves. Control is in brown (7F6000). The percentage is calculated by comparing the intensity in the test group to the positive control. Red asterisks (*) show the significance of the level of difference between α-Syn and α-Syn aggregates grown in the presence of lipids as well as between lipid samples and α-Syn. Blue asterisks show the significance of the level of difference between α-Syn aggregates formed in PC and PS conditions. NS is a nonsignificant difference, and *p ≤ 0.05, **p ≤ 0.01, and ***p ≤ 0.001.

We investigated toxicity α-Syn aggregates formed at the lag and growth phases, as well as α-Syn aggregates formed at the stage of protein aggregation, Figure 5. Amyloid aggregates exert toxicities by enhancing ROS production, simultaneously despairing mitochondrial activity in cells.^29,57^ Therefore, we examined the extent to which α-Syn aggregates are engaged in reactive oxygen species (ROS) production and mitochondrial dysfunction in N27 rat neuron cells. In addition to the ROS production assay which measures the oxidative stress level in the neurons, we also performed the JC-1 assay and the lactate dehydrogenase (LDH) assay. The JC-1 assay monitors the mitochondrial membrane potential level to detect mitochondrial dysfunction.^58^ JC-1 can interconvert between the monomer and the aggregated form based on the potential. Lactate dehydrogenase catalyzes the reaction of converting lactate into pyruvate, the glycolysis process.^59^ When the cell is damaged or dead, this enzyme LDH will be released into the extracellular environment.

The ROS test showed that the lag-phase α-Syn aggregates grown in the presence of both PC and PS induced significantly higher levels of oxidative stress compared to α-Syn grown in a lipid-free environment, Figure 5, top. It should be noted that the toxicity of α-Syn:PC and α-Syn:PS oligomers was found to be similar to each other. At the same time, α-Syn:PC and α-Syn:PS aggregates formed at the growth phase produced distinctly different ROS levels. Specifically, α-Syn:PC oligomers exerted higher levels of ROS compared α-Syn oligomers and lower than α-Syn:PS aggregates. It should be noted that both oligomeric and fibrillar species are present at this stage of protein aggregation in α-Syn and α-Syn:PS samples. Finally, ROS levels caused by both α-Syn:PS and α-Syn:PC aggregates observed at the late stage of protein aggregation were similar to each other but higher than the ROS exerted by α-Syn grown in a lipid-free environment. It should be noted that lipids themselves exerted insignificant levels of ROS.

The JC-1 assay confirmed that α-Syn:PS aggregates formed at all time points of protein aggregation were more toxic to mitochondria than α-Syn:PC and α-Syn aggregates, Figure 5, middle. We also found that the levels of mitochondrial dysfunction caused by α-Syn:PC and α-Syn oligomers grown at the lag phase and late stage of protein aggregation were similar to each other. It should be noted that a significant difference between all the three groups of aggregates formed at the middle stage of protein aggregation was observed. Specifically, α-Syn:PS caused a higher level of mitochondrial dysfunction compared to α-Syn:PC oligomers that, in turn, were more toxic to N27 cells than α-Syn aggregates. It should be noted that lipids themselves did not cause any mitochondrial dysfunction.

The LDH assay showed that protein oligomers formed at the early stage of α-Syn aggregation exerted distinctly different levels of toxicity, Figure 5, bottom. Specifically, α-Syn oligomers were significantly less toxic than α-Syn:PC, which, in turn, exerted lower cell toxicity than α-Syn:PS oligomers. The same trend was preserved for the protein aggregates grown at the middle phase and late stage of protein aggregation. Finally, we want to point out that lipids themselves were significantly less toxic than lipid/protein aggregates. These results show that lipids determine the toxicity of α-Syn aggregates that were formed in their presence. Specifically, both PC and PS increased the toxicity of α-Syn aggregates, whereas the effect of PS is much greater than the effect caused by PC.

One can expect that the lipid-determined changes in the toxicity of α-Syn oligomers and fibrils originate from (i) the above-discussed differences in the secondary structure of these aggregates and (ii) the presence of PC and PS in these aggregates. We infer that changes in the secondary structure of oligomers and fibrils arise from electrostatic and hydrophobic interactions that are taken place between lipids and α-Syn. Using NMR and fluorescence, Viennet and co-workers found that headgroups of phospholipids interacted with lysine and glutamic acid residues on the N-terminus (aa 1–60) of a-Syn. In parallel, the fatty acids of lipids established hydrophobic interactions with the central domain (aa 61–95) of α-Syn, also known as NAC domain.^60−62^ Thus, the presence of such lipid–protein complexes at the stage of oligomer nucleation results in a distinctly different protein secondary structure of α-Syn:PC and α-Syn:PS compared to the α-Syn aggregates grown in the lipid-free environment. One can also expect that the presence of PC and PS in the structure of α-Syn:PC and α-Syn:PS aggregates enhances their membrane permeability properties. Thus, such lipids facilitate oligomer accumulation in cells where they damage mitochondria and enhance ROS production.

## Conclusions

Summarizing, our experimental finding shows that PC and PS uniquely alter the rates of α-Syn aggregation. PS strongly accelerated α-Syn fibril formation, whereas PC inhibited α-Syn fibril formation. Furthermore, we found that both PC and PS uniquely altered the secondary structure of α-Syn oligomers which are fibrils formed at the early, middle, and late stages of protein aggregation. These changes in the secondary structures, as well as the presence of both PS and PC in the oligomers and fibrils, enhanced the toxicity of these protein aggregates to mice midbrain N27 cells. These findings suggest that lipids play an important role in the aggregation of α-Syn, determining the toxicity of α-Syn oligomers and fibrils.

## Materials and Methods

### Protein and Lipid Preparation

α-Syn is purchased from AnaSpec, CA, USA. The preparation of α-synuclein is followed by the group protocol by Zhou and Kurouski. α-Syn was dissolved to a final concentration of 150 μM in 1× PBS buffer, pH at 7.4 stock. Next, the stock was mixed with DMPC or DMPS lipid unilamellar vesicles (LUVs) and reached the final protein concentration at 45 μM. The aggregation took place under 37 °C and 510 rpm in the plate reader.

The preparation of lipid unilamellar vesicles (LUVs) was repeated using the previous protocol by Dou and Kurouski. After collecting LUVs through the extruder, the sizes of lipid LUVs were checked using dynamic light scattering (DLS). In this experiment, we investigate the protein-to-lipid ratio 1:2 samples for AFM-IR and kinetics.

### Kinetic Measurements

α-Syn aggregation was monitored using thioflavin T (ThT) fluorescence assay on a plate reader (Infinite M200, Tecan) under 37 °C and 510 rpm at pH 7.4. α-Syn was mixed with lipid LUVs in the 1:2 P/L ratio; the final concentration is 150 and 300 μM, respectively. The protein solution was mixed with Th-T to reach the final concentration of ThT equal to 25 μM. A total volume of 120 μL solution was loaded into wells for kinetic measurement. Excitation was 450 nm; the emission signal was collected at 490 nm. The final kinetic curve (Figure S1) was averaged by three repeats.

### AFM-IR Imaging and Spectroscopy

The solution (3–6 μL) of aggregate samples was deposited on a silicon wafer and exposed for 5–10 min for drying. Next, the excess samples were removed, rinsed with DI water, and dried under a N_2_ flow. AFM-IR imaging was conducted using a Nano-IR3 system (Bruker, Santa Barbara, CA, USA). The IR source was a QCL laser. Contact-mode AFM tips (ContGB-G AFM probe, NanoAndMore) were used to obtain all spectra and maps. No evidence of the sample distortion was observed upon contact-mode AFM imaging. IR maps at 1624, 1655, and 1694 cm^–1^ wavenumber values were obtained to study the secondary structure of α-Syn:PS and α-Syn:PC oligomers. Phase loop lock was enabled during the mapping with a 0.03 V threshold. iGain and pGain vary based on different particle heights, but in general from 0.5 to 1 for iGain and 1 to 2 for pGain. The scan rate is 0.8 Hz, and the resolution *X* and *Y* is 512 pts. AFM height and deflection images were acquired simultaneously with IR maps. 20 point measurements were taken from every analyzed oligomer and individual aggregates (Figures S5–S7). The spectra were zapped from 1648 to 1652 cm^–1^ due to chip-to-chip transition artifact from the instrument. The spectral resolution is 2 cm^–1^/pt. Savitzky–Golay smoothing was applied to all spectra with two polynomial orders by using MATLAB.

### Cell Toxicity Assay

Mice midbrain N27 cells, a model cell line for Parkinson’s disease, were grown in RPMI 1640 medium (Thermo Fisher Scientific, Waltham, MA, USA) with 10% fetal bovine serum (FBS) (Invitrogen, Waltham, MA, USA) in a 96-well plate (5000 cells per well) at 37 °C under 5% CO_2_. After 24 h, the cells were found to fully adhere to the wells reaching ∼70% confluency. Next, 100 μL of the cell culture was replaced with 100 μL of RPMI 1640 medium with 5% FBS-containing protein samples. After 48 h of incubation with the sample of the protein aggregates, a lactate dehydrogenase (LDH) assay was performed on the cell medium using the CytoTox 96 non-radioactive cytotoxicity assay (G1781, Promega, Madison, WI, USA). For the positive control, 10 μL of lysis buffer provided in G1781, Promega, Madison, WI, USA, was added to the cells. Absorption measurements were made in a plate reader (Tecan, Männedorf, Switzerland) at 490 nm. All experiments were done in triplicates. Every well was measured 25 times in different locations.

In parallel, reactive oxygen species (ROS) assay was performed using the same cell culture. Briefly, ROS reagent (C10422, Invitrogen, Waltham, MA, USA) was added to reach the final concentration of 5 μM and incubated at 37 °C under 5% CO_2_ for 30 min. After the supernatant was removed, the cells were washed with PBS and resuspended in 200 μL of PBS in the flow cytometry tubes. For the positive control, menadione was used. Cells were incubated with menadione for 30 min prior to measurements. Sample measurements were made in an Accuri C6 Flow Cytometer (BD, San Jose, CA, USA) using a red channel (λ = 633 nm). Percentages of ROS cells were determined using Acura software.

For JC-1 staining, 1 μL of JC-1 reagent (M34152A, Invitrogen) was added to cells and incubated at 37 °C under 5% CO_2_ for 30 min. After the supernatant was removed, the cells were washed with PBS and resuspended in 200 μL of PBS in the flow cytometry tubes. For the positive control, carbonyl cyanide chlorophenylhydrazone was used. Cells were incubated with menadione for 5 min prior to measurements. Sample measurements were made in an Accuri C6 Flow Cytometer (BD, San Jose, CA, USA) using a red channel (*k* = 633 nm). The percentages of ROS cells were determined using ACCURI software.

For both LDH and ROS controls, PBS (pH 7.4) was added to cells and incubated under the same experimental conditions. T-test was used to analyze the results: *p ≤ 0.05, **p ≤ 0.01, and ***p ≤ 0.001 relative to untreated cells are shown. NS indicates “non-significant” differences between the toxicity or ROS response of the samples.

## Supporting Information Available

Detailed description of spectra acquisition methods and instrumentation; ThT kinetic curve; AFM images; histograms; height profiles; AFM-IR spectra; AFM-IR deconvolution spectra; and T-test results (PDF)
