# Supplementary material for: Elucidation of Secondary Structure and Toxicity of α-Synuclein Oligomers and Fibrils Grown in the Presence of Phosphatidylcholine and Phosphatidylserine
Source: ACS Chem Neurosci. 2023 Aug 21;14(17):3183–91. doi: 10.1021/acschemneuro.3c00314 (PMC10862479; doi:10.1021/acschemneuro.3c00314)
Supplement: Supplementary file 1 — cn3c00314_si_001.pdf [file cn3c00314_si_001.pdf]

Elucidation of Secondary Structure and Toxicity of Alpha-Synuclein Oligomers and Fibrils Grown in the Presence of Phosphatidylcholine and Phosphatidylserine

Tianyi Dou[1†] Mikhail Matveyenka[1†], and Dmitry Kurouski[*1,2]

1. Department of Biochemistry and Biophysics, Texas A&M University, College Station, Texas 77843, United States

2. Department of Biomedical Engineering, Texas A&M University, College Station, Texas, 77843, United States

Dmitry Kurouski

Email: dkurouski@tamu.edu Tel: 979-458-3778
ORCID
Dmitry Kurouski: 0000-0002-6040-4213

Supporting Information

Table S1—page 2. T-test of protein secondary structures for α-Syn aggregates in lipid-free condition

Table S2—page 2. T-test of protein secondary structures for α-Syn aggregates in DMPS LUVs

Table S3—page 2. T-test of protein secondary structures for α-Syn aggregates in DMPC LUVs

Figure S1—page 3. ThT kinetic curve of α-Syn aggregation

Figure S2—page 3. AFM images and histograms of high distributions of α-Syn aggregates

Figure S3—page 4. AFM images and histograms of high distributions of α-Syn:PC aggregates

Figure S4—page 4. AFM images and histograms of high distributions of α-Syn:PS aggregates

Figure S5—page 5. AFM images, height profile, and AFM-IR spectra of α-Syn aggregates

Figure S6—page 6. AFM images, height profile, and AFM-IR spectra of α-Syn:PC aggregates

Figure S7—page 7. AFM images, height profile, and AFM-IR spectra of α-Syn:PS aggregates

Figure S8—page 8. Histograms of the amount of different protein secondary structures in α-Syn, α-Syn: PC, and α-Syn: PS aggregates

Figure S9—page 9. A histogram of the amount of different protein secondary structures in α-Syn aggregates

Figure S10—page 10. A histogram of the amount of different protein secondary structures in α-Syn:PS aggregates

Figure S11—page 11. A histogram of the amount of different protein secondary structures in α-Syn:PC aggregates

Figure S12—page 12. The AFM-IR deconvolution spectra of α-Syn, α-Syn:PC, and α-Syn:PS

Table S1. T-test of protein secondary structure in α-Syn aggregates grown in the lipid-free environment. NS: no significant difference; *$P \leq 0.05$; **$P \leq 0.01$; ***$P \leq 0.001$

		P-value	
Parallel Beta Sheet	Lag to Growth	0.018	*
	Lag to Fibril	0.203	NS
	Growth to Fibril	0.024	*
Alpha Helix	Lag to Growth	0.018	*
	Lag to Fibril	0.357	NS
	Growth to Fibril	0.026	*
Turns	Lag to Growth	0.545	NS
	Lag to Fibril	0.546	NS
	Growth to Fibril	1	NS
Anti-parallel Beta Sheet	Lag to Growth	0.054	*
	Lag to Fibril	0.052	*
	Growth to Fibril	0.283	NS

Table S2. T-test of protein secondary structure in α-Syn aggregates grown in the presence of DMPS LUVs (protein: lipid 1:2 ratio). NS: no significant difference; *$P \leq 0.05$; **$P \leq 0.01$; ***$P \leq 0.001$

		P-value	
Parallel Beta Sheet	Lag to Growth	0.827	NS
	Lag to Fibril	0.417	NS
	Growth to Fibril	0.014	*
Alpha Helix	Lag to Growth	0.018	*
	Lag to Fibril	0.050	*
	Growth to Fibril	0.046	*
Turns	Lag to Growth	0.887	NS
	Lag to Fibril	0.023	*
	Growth to Fibril	0.0001	***
Anti-parallel Beta Sheet	Lag to Growth	0.0038	**
	Lag to Fibril	0.0128	*
	Growth to Fibril	.0005	**

Table S3. T-test of protein secondary structure in α-Syn aggregates grown in the presence of DMPC LUVs (protein: lipid 1:2 ratio). NS: no significant difference; *$P \leq 0.05$; **$P \leq 0.01$; ***$P \leq 0.001$

		P-value	
Parallel Beta Sheet	Early to mid	0.0002	***
	Early to Late	0.0003	***
	Mid to Late	0.455	NS
Alpha Helix	Early to mid	0.1116	NS
	Early to Late	0.0561	NS
	Mid to Late	0.480	NS
Turns	Early to mid	0.017	*
	Early to Late	0.005	**

	Mid to Late	0.760	NS
Anti-parallel Beta Sheet	Early to mid	0.135	Ns
	Early to Late	0.001	***
	Mid to Late	0.506	NS

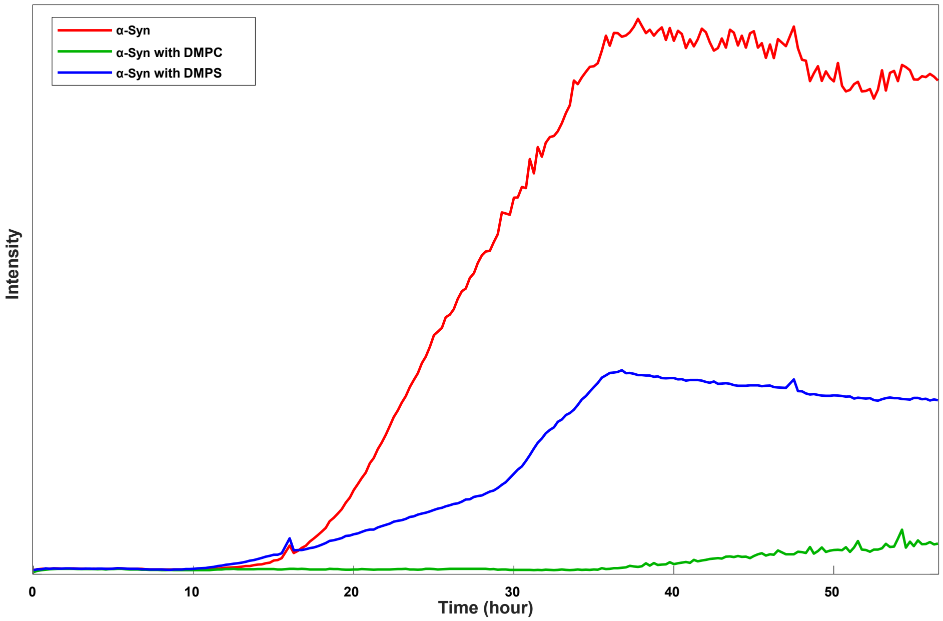

Figure S1. α-Syn aggregation kinetics by thioflavin Th-T assay at 37°C, 510 RPM.

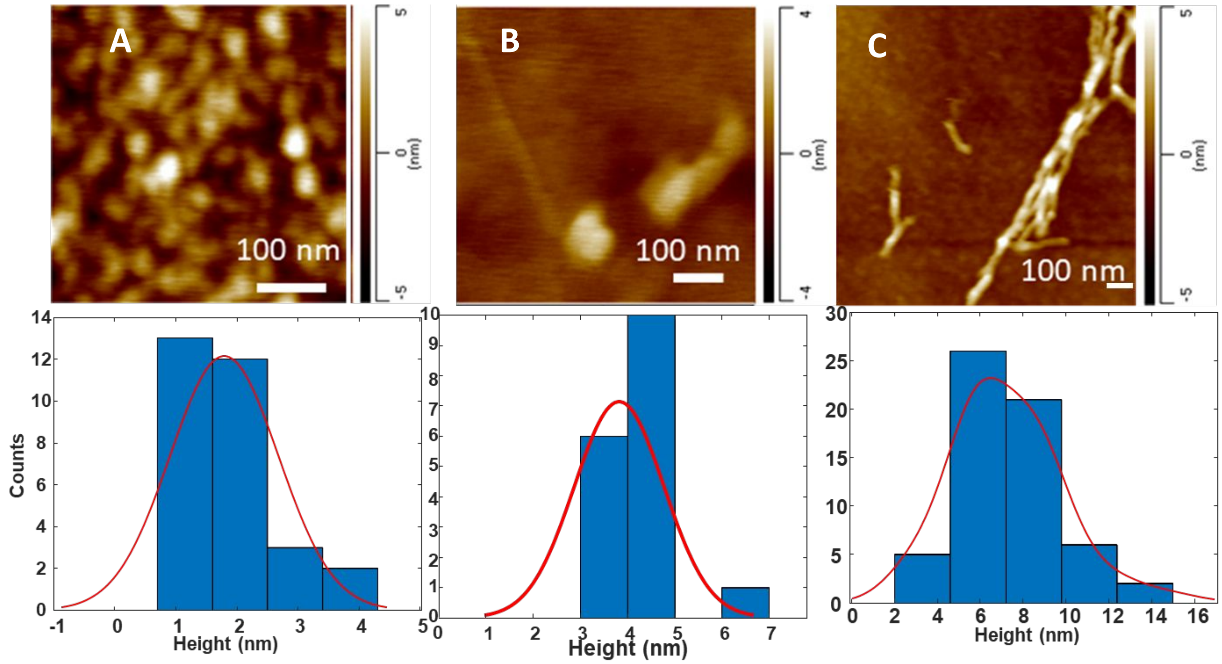

Figure S2. AFM images and histograms of high distributions of α-Syn aggregates formed at 20 h (A) 32 h (B) and 50 h (C) of protein aggregation in the lipid-free conditions.

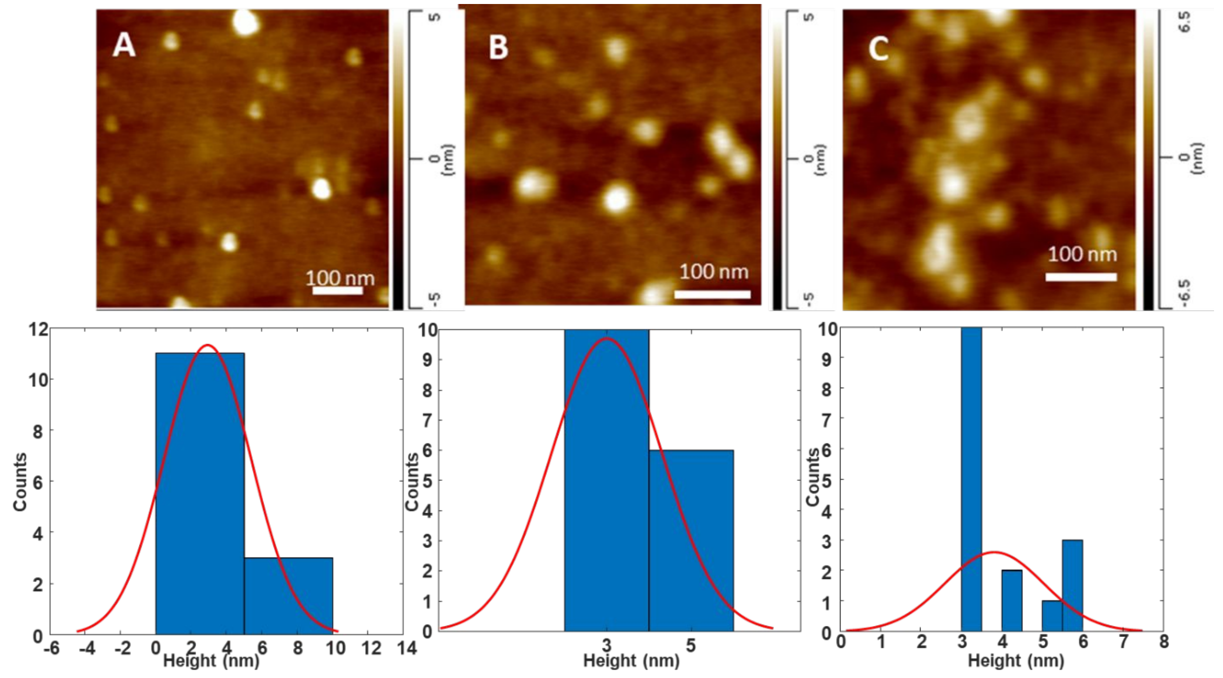

Figure S3. AFM images and histograms of high distributions of α-Syn:PC aggregates formed at 20 h (A) 32 h (B) and 50 h (C) of protein aggregation in the presence of DMPC LUVs (P:L ratio = 1:2).

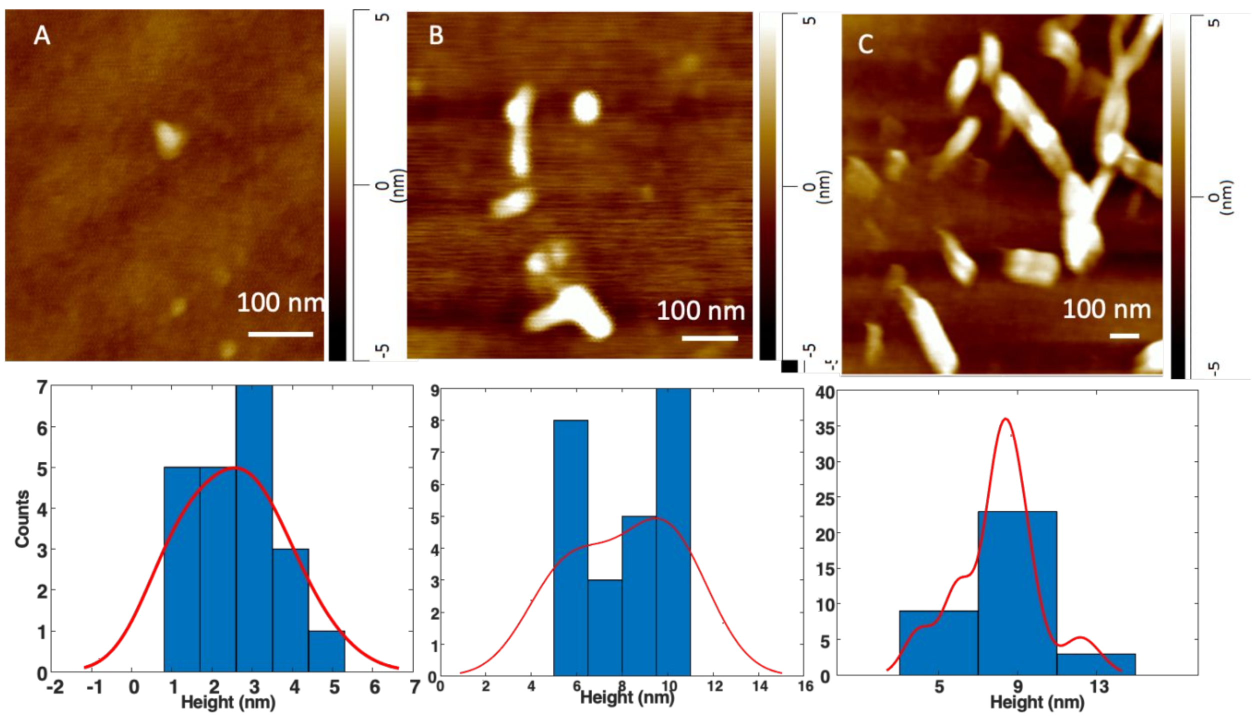

Figure S4. AFM images and histograms of high distributions of α-Syn:PS aggregates formed at 20 h (A) 32 h (B) and 50 h (C) of protein aggregation in the presence of DMPS LUVs (P:L ratio = 1:2).

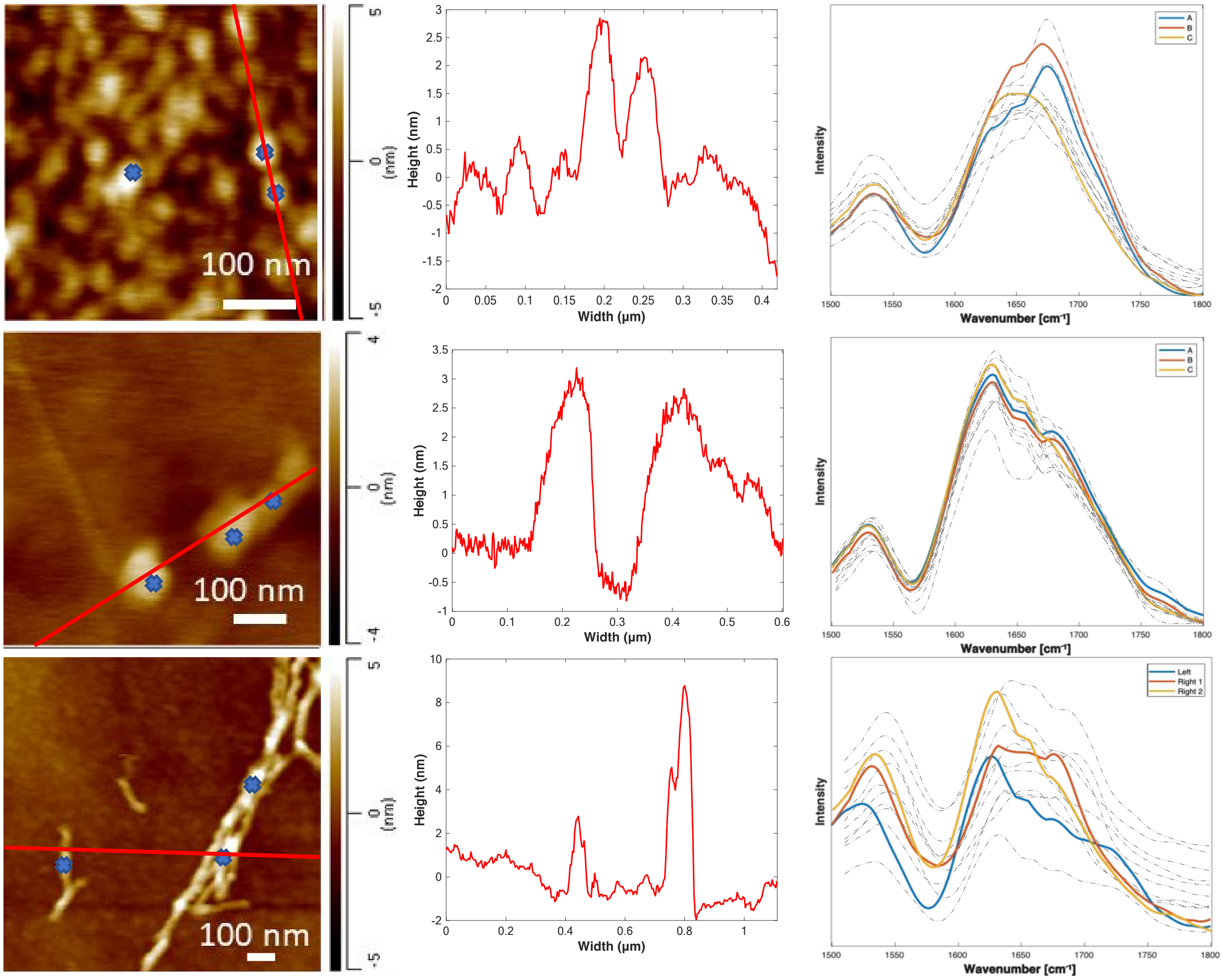

Figure S5. AFM images (left panels) with height profiles (middle panels) of α-Syn aggregates formed at 20 h (A) 32 h (B) and 50 h (C) of protein aggregation in the lipid-free conditions. Averaged (colored solid) and individual (dashed grey) AFM-IR spectra (right panels) collected at labeled by blue asterisk locations.

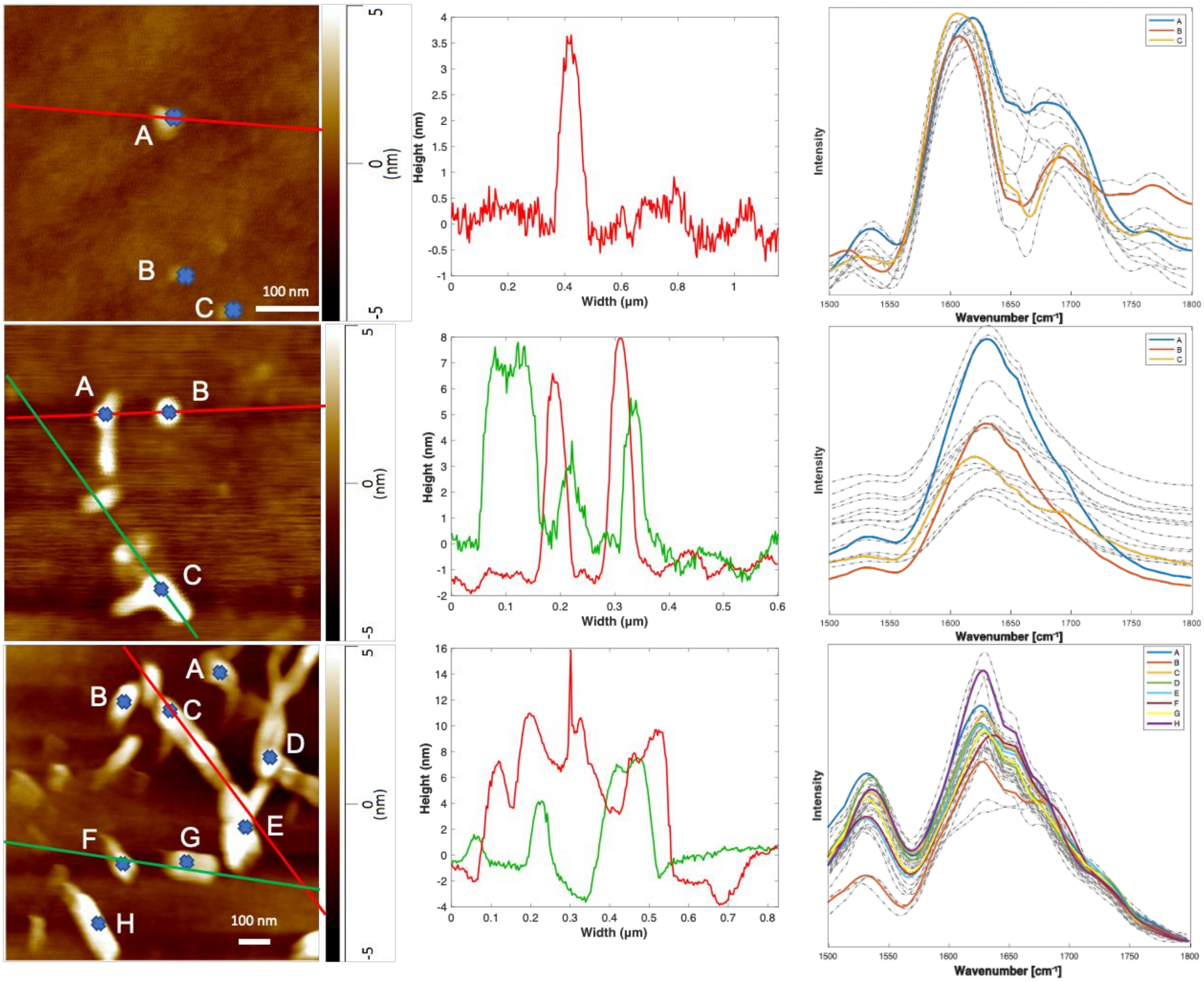

Figure S6. AFM images (left panels) with height profiles (middle panels) of α-Syn:PS aggregates formed at 20 h (A) 32 h (B) and 50 h (C) of protein aggregation in the presence of DMPS LUVs (protein: lipid 1:2 ratio). Averaged (colored solid) and individual (dashed grey) AFM-IR spectra (right panels) collected at labeled by blue asterisk locations.

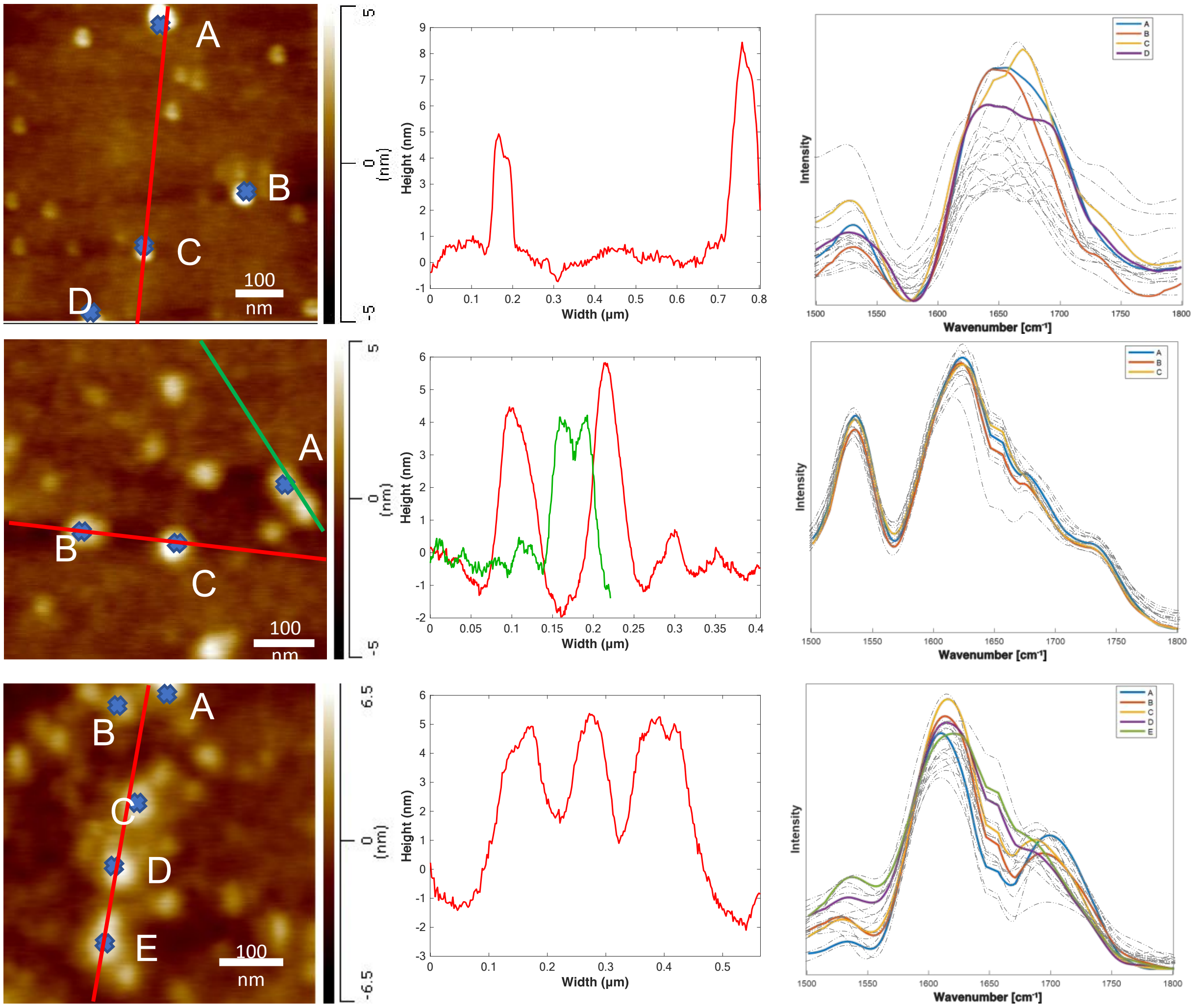

Figure S7 AFM images (left panels) with height profiles (middle panels) of α-Syn: PC aggregates formed at 20 h (A) 32 h (B) and 50 h (C) of protein aggregation in the presence of DMPC LUVs (protein: lipid 1:2 ratio). Averaged (colored solid) and individual (dashed grey) AFM-IR spectra (right panels) collected at labeled by blue asterisk locations.

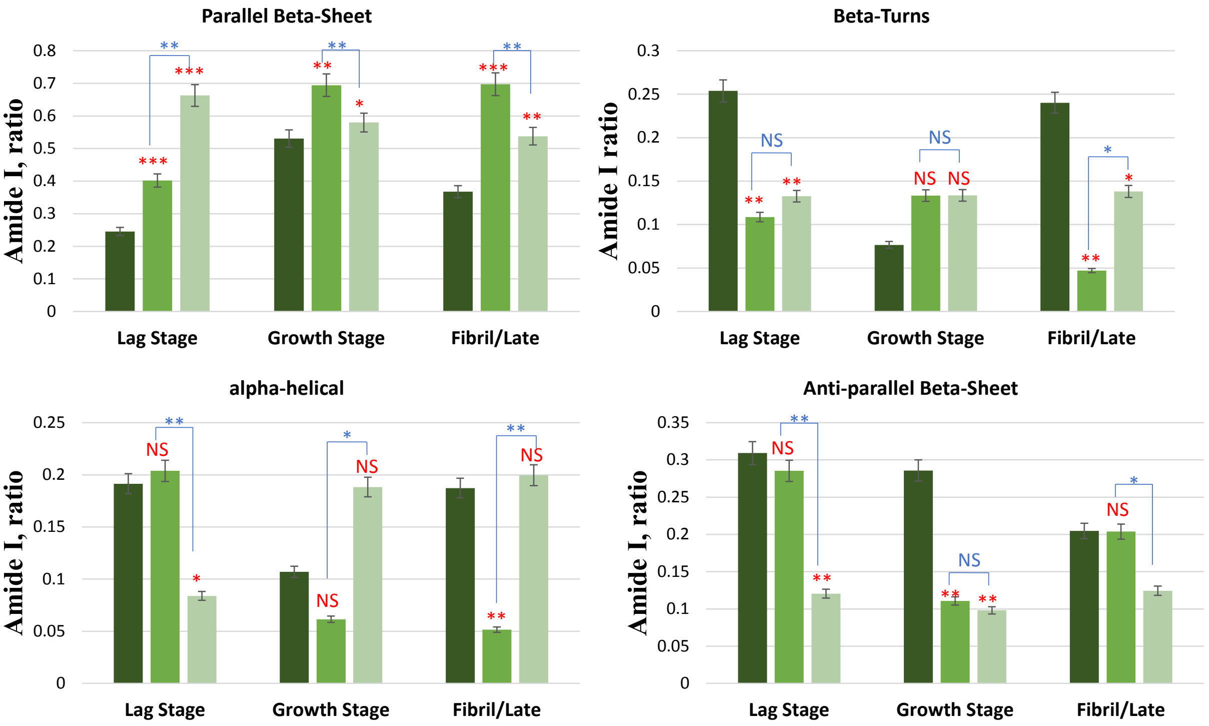

Figure S8. Histograms of the amount of different protein secondary structures in α-Syn (olive green), α-Syn: PC (green), and α-Syn: PS (light green) aggregates at 20 h (lag stage), 32 h (growth stage) and at 50 h (the plateau of protein aggregation) according to the fitting of AFM-IR spectra acquired from these protein species. NS: no significant difference; *$P \leq 0.05$; **$P \leq 0.01$; ***$P \leq 0.001$.

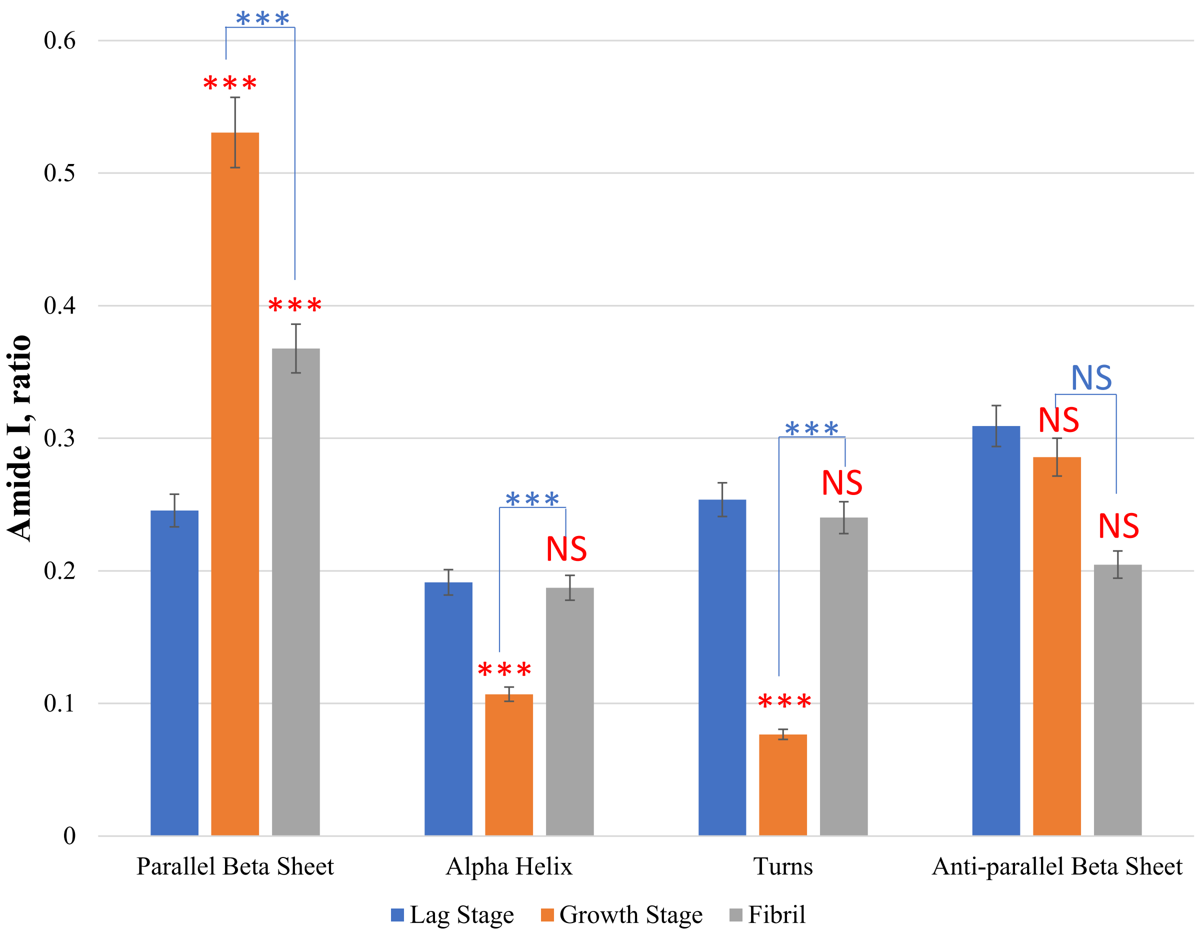

Figure S9. A histogram of the amount of different protein secondary structures in α-Syn aggregates formed at different stages of protein aggregation in the lipid-free environment. NS: no significant difference; *$P \leq 0.05$; **$P \leq 0.01$; ***$P \leq 0.001$

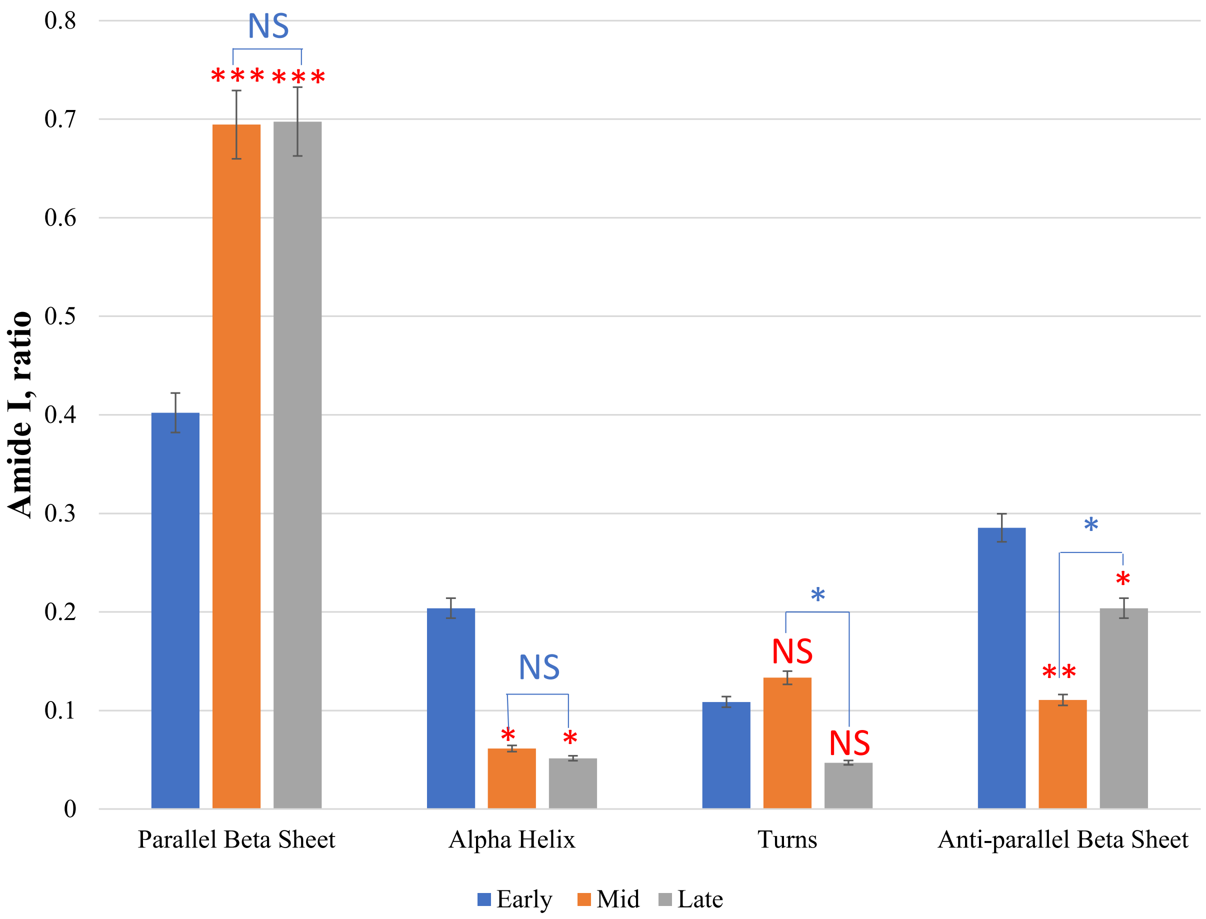

Figure S10. A histogram of the amount of different protein secondary structures in α-Syn aggregates formed at different stages of protein aggregation in the presence of DMPS LUVs (protein: lipid 1:2 ratio). NS: no significant difference; *$P \leq 0.05$; **$P \leq 0.01$; ***$P \leq 0.001$

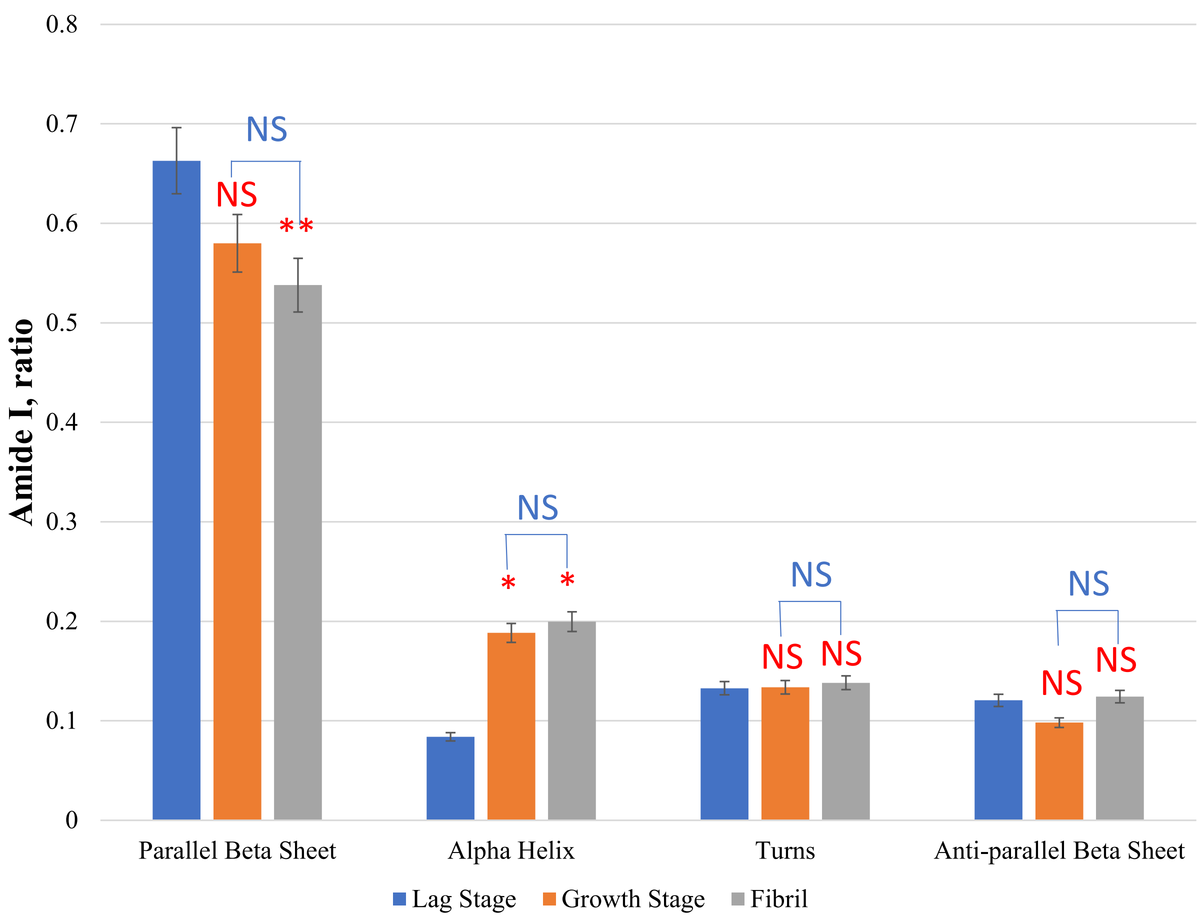

Figure S11. A histogram of the amount of different protein secondary structures in α-Syn aggregates formed at different stages of protein aggregation in the presence of DMPC LUVs (protein: lipid 1:2 ratio). NS: no significant difference; *$P \leq 0.05$; **$P \leq 0.01$; ***$P \leq 0.001$

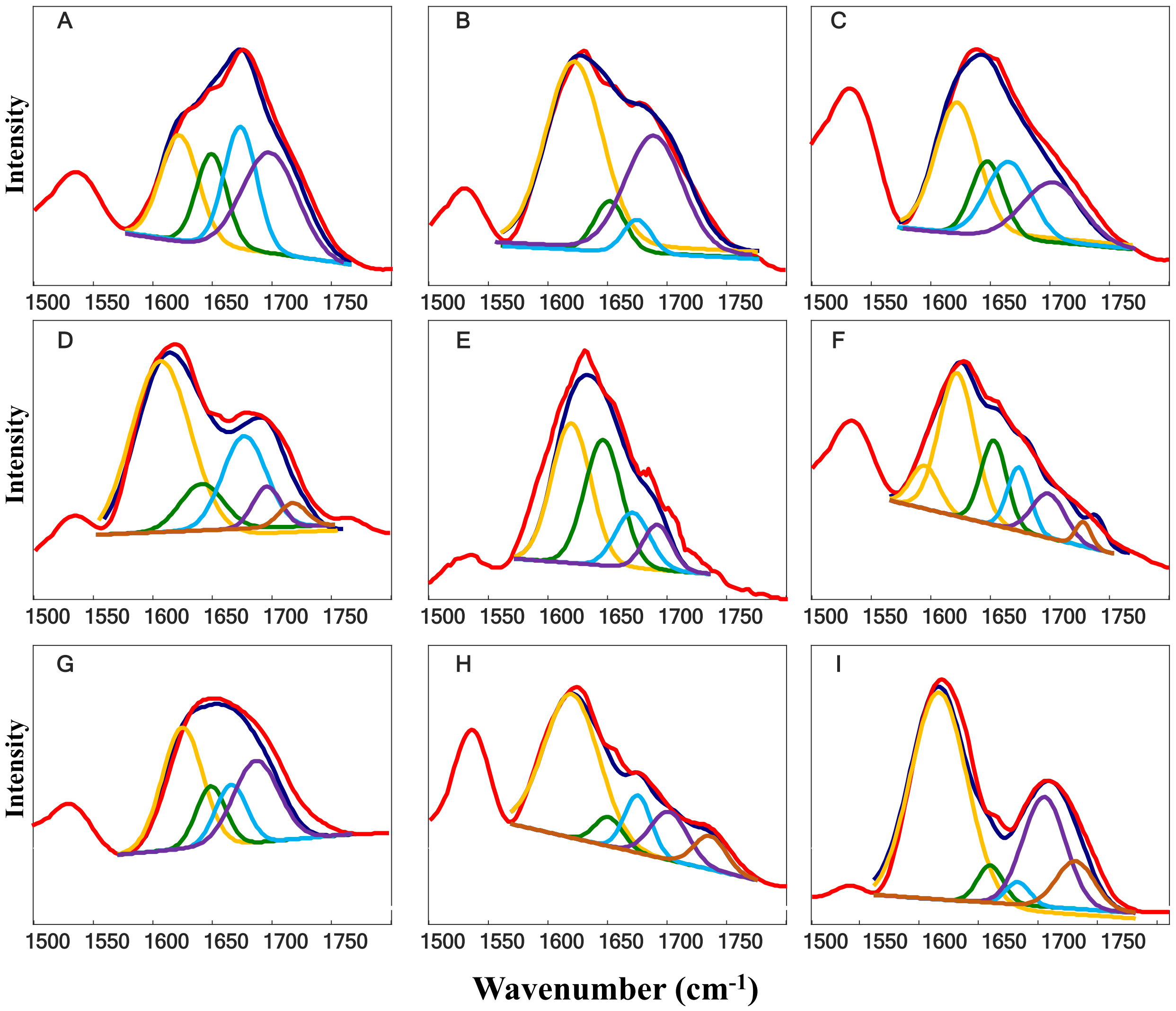

Figure S12. The AFM-IR deconvolution spectra of α-Syn in lipid-free conditions (A-C), in DMPS LUVs (D-F), and DMPC LUVs (G-I). Original spectra in red, fitting spectra in navy blue. The parallel beta-sheet is in yellow, the alpha-helix in green turns in light blue, and the anti-parallel beta-sheet is in purple, with lipid in orange.
